# A multi-centric Study of *Candida* bloodstream infection in Lima-Callao, Peru: Species distribution, antifungal resistance and clinical outcomes

**DOI:** 10.1371/journal.pone.0175172

**Published:** 2017-04-18

**Authors:** Lourdes Rodriguez, Beatriz Bustamante, Luz Huaroto, Cecilia Agurto, Ricardo Illescas, Rafael Ramirez, Alberto Diaz, Jose Hidalgo

**Affiliations:** 1 Guillermo Almenara Hospital, Lima, Peru; 2 Instituto de Medicina Tropical Alexander von Humboldt, Cayetano Heredia University, Lima, Peru; 3 Dos de Mayo Hospital, Lima, Peru; 4 Alberto Sabogal Hospital, Callao, Peru; Institute of Microbiology, SWITZERLAND

## Abstract

**Background:**

The incidence of candidemia is increasing in developing countries. Very little is known about the epidemiology of candidemia in Peru. The aim of this study is to describe the incidence, microbiology, clinical presentation and outcomes of *Candida* bloodstream infections in three Lima-Callao hospitals.

**Methods:**

*Candida* spp. isolates were identified prospectively at participant hospitals between November 2013 and January 2015. Susceptibility testing for amphotericin B, fluconazole, posaconazole, voriconazole and anidulafungin was performed using broth microdilution method. Clinical information was obtained from medical records and evaluated.

**Results:**

We collected information on 158 isolates and 157 patients. Median age of patients was 55.0 yrs., and 64.1% were males. Thirty-eight (24.2%) episodes of candidemia occurred in those <18 yrs. The frequency of non-*Candida albicans* was 72.1%. The most frequently recovered species were *C*. *albicans* (n = 44, 27.8%), *C*. *parapsilosis* (n = 40, 25.3%), *C*. *tropicalis* (n = 39, 24.7%) and *C*. *glabrata* (n = 15, 9.5%). Only four isolates were resistant to fluconazole, 86.7% (n = 137) were susceptible and 17 were susceptible-dose dependent. Decreased susceptibility to posaconazole was also observed in three isolates, and one to voriconazole. All isolates were susceptible to anidulafungin and amphotericin B. The most commonly associated co-morbid conditions were recent surgery (n = 61, 38.9%), mechanical ventilation (n = 60, 38.2%) and total parenteral nutrition (n = 57, 36.3%). The incidence of candidemia by center ranged between 1.01 and 2.63 cases per 1,000 admissions, with a global incidence of 2.04. Only 28.1% of cases received treatment within 72 hrs. of diagnosis. Overall, the 30-day survival was 60.4% (treated subjects, 67.4%; not-treated patients, 50.9%).

**Conclusions:**

We found a very high proportion of non-*albicans Candida* species. Despite this, the decreased susceptibility/resistance to fluconazole was only 13.3% and not seen in the other antifungals. Overall, the incidence of candidemia mortality was high when compared to other international studies. It is possible, that the delay in initiating antifungal treatment contributed to the elevated mortality rate, in spite of low antifungal resistance.

## Introduction

*Candida* species are amongst the most frequent pathogens in nosocomial bloodstream infections. In recent years, information from different countries around the world have consistently shown this trend. Several studies recently published from Latin American countries, [1–3] including one report with Peruvian data from nine hospitals, [4] show that candidemia is a significant cause of bloodstream infection in the region.

Candidemia is associated with high mortality, has a non-specific clinical presentation and a high index of suspicion is required for a timely diagnosis and initiation of therapy. In a low-resource setting, dealing with complicated patients with multiple co-morbidities and multiple medical interventions, diagnosis presents additional challenges. Low awareness amongst clinicians and a lack of regular access to diagnostic resources such as blood cultures are important factors that may contribute to the high mortality observed in our patients. [5]

In Latin American countries access to more complex medical care over the past few decades has been associated with new hospital acquired infectious complications. The two most common complications include multidrug resistant nosocomial infections and invasive mycoses. However, since candidemia is not recognized as a frequent cause of sepsis, and clinicians have a low index of suspicion, candidemia tends to be lower on the differential diagnosis list and becomes a consideration as a cause of sepsis much later and thus the initiation of appropriate antifungal therapy is delayed.

Several studies estimating the incidence of candidemia in Northern hemispheric countries [6–10] report rates that are consistently lower than the ones reported in Latin American countries. [2, 11] A prior report that included five Latin American countries (Argentina, Brazil, Colombia, Mexico, and Peru) showed that the incidence of device-associated infectious complications in lower resources countries ICUs was higher than rates found in the United States. [12]

An internal report from Almenara Hospital in Peru for the years between 2009–2010 found that *Candida* species was the most frequently isolated organism from blood cultures in the General ICU [13] and for the period 2012–2014 it was the 2^nd^ most commonly recovered isolate after coagulase-negative staphylococci. [14]

The aim of this study is to describe the characteristics of *Candida* bloodstream infections evaluating the frequency, species distribution, *in vitro* susceptibility patterns, clinical presentation, associated co-morbidity conditions, and outcomes, in three Peruvian hospitals, located in Lima-Callao.

## Material and methods

Study was approved at each participating Hospital by its corresponding Research Committee on the following dates: Comité de Investigación Hospital Sabogal, Approval letter 11 April 2012; Comité de Ética en Investigación Hospital Guillermo Almenara, Approval letter 12 September 2012; Oficina de Apoyo a la Investigación Hospital Dos de Mayo, Approval letter 15 August 2013. Informed consent was waived since the data was de-identified and the specimens were obtained as part of the routine clinical care. The institutional review boards of each hospital approved the original study protocol and the forms to collect data.

### Study design

This was a prospective study performed at three tertiary care hospitals in Lima-Callao, Peru. Participating hospitals were Guillermo Almenara Hospital, Dos de Mayo Hospital in Lima, and Alberto Sabogal Hospital in Callao. Lima and Callao are part of the same metropolitan area and share geographical and epidemiological characteristics.

While Almenara (818 beds) and Sabogal (408 beds) Hospitals are Social Security hospitals, and Dos de Mayo Hospital (602 beds) is a Ministry of Health hospital. These two networks (Social Security and Ministry of Health) are the major public institutions providing health care in the country. The proportion of ICU beds to general wards in each hospital is low, in a range of 8–10%, and thus many complex patients are taken care of on the general wards. Almenara is the only hospital in the group with transplantation programs.

Prospective laboratory-based surveillance was conducted between November 2013 and January 2015. All *Candida* species recovered from blood samples were included the study.

Age ranges were defined as neonates: ≤28 days old or staying in NICU; children: age ≥ 28 days up to 18 years; adults: ages between 19 and 60 years; elderly: ≥ 60 years. Patients’ medical records were reviewed and relevant information was included in the study database (clinical presentation, demographic information, associated conditions, treatment received, and outcomes).

We performed a survival analysis using the information collected in clinical records of patients with a diagnosis of candidemia.

### Laboratory procedures

All isolates were initially recovered by the microbiology laboratory from each hospital. Conventional microbiological methods were used at Sabogal and Dos de Mayo Hospitals. An automated system Vitek 2 was used at Almenara Hospital when available.

Isolates were then sent to the Clinical Mycology Laboratory at the Universidad Peruana Cayetano Heredia for microbiological and molecular verification of species identification and susceptibility testing. Laboratory methods are described in detail elsewhere [4].

CHROMagarTM *Candida* medium (Difco, USA) was used to evaluate the colony purity and viability and for the presumptive identification of *Candida* species. Isolates were preserved using both distilled water at room temperature and yeast extract-peptone-dextrose broth medium (YEPD, contained 1% Yeast extract, 2% Peptone and 2% Dextrose) at 4–8°C.

Species identification was determined using standard methods such as microscopic morphology on corn meal agar+Tween 80, growth at 37°C and 42°C, growth in hypertonic medium, and the API 20 C AUX system (bioMérieux, Marcy-l’Étoile, France). In addition, *C*. *albicans*, *C*. *parapsilosis* species complex and *C*. *glabrata* were identified using sequencing of the ITS ribosomal DNA region by Macrogen (USA) and compared to sequence data available from GenBank. The sequences generated in this study have been deposited in the GenBank database under accession numbers: KY593144 to KY593164, KY604744 to KY604763, KY608058 to KY608075, KY611779 to KY611800 and KY619289 to KY619304.

Susceptibility testing for amphotericin B, fluconazole, posaconazole, voriconazole and anidulafungin was performed using CLSI broth microdilution method. MIC breakpoints were interpreted according to the CLSI M27-S3 and M27-S4 documents, as well as the new CLSI species-specific clinical breakpoints for fluconazole, voriconazole and echinocandins.[15–17] Since accepted interpretive breakpoints have not been established for amphotericin B, we used the epidemiological cut-off value (ECV) to categorize an isolate as WildType (WT) (≤2 mg/L) or non-WT (>2 mg/L) and differentiate an isolate with reduced susceptibility.[18] Isolates of *C*. *albicans*, *C*. *tropicalis* and *C*. *parapsilosis* with MICs of ≥ 8 μg ml^-1^ and isolates of *C*. *glabrata* with MICs of ≥ 64 μg ml^-1^ were considered resistant to fluconazole. Anidulafungin is conventionally used for assessment of susceptibility to echinocandins due to its in vitro stability. For posaconazole, ECV were used to identify reduced susceptibility (MIC>0.06). [19] Fluconazole, voriconazole and anidulafungin (Pfizer) were used for susceptibility testing.

### Data analysis

Descriptive statistics included frequencies, percentages and means with standard deviation. We performed a bivariate and multiple Cox regression analysis to determine association between risk factors (antifungal treatment, severity of episode, *Candida* species, and presence of severe sepsis) and the mortality risk, controlling this relationship by age and sex of subjects. We chose the best model based on the likelihood ratio test. A p-value of <0.05 was taken to indicate statistical significance. All analyses were conducted using Stata version 12 (StataCorp LP).

## Results

A total of 158 *Candida* strains were isolated during the study period. Of these, 99 (62.7%) came from Almenara Hospital, 30 (19.0%) came from Dos de Mayo Hospital, and 29 (18.3%) came from Sabogal Hospital.

The overall incidence was 2.04 cases per 1000 admissions. The incidence was highest for Almenara Hospital with 2.63 and lowest for Dos de Mayo Hospital with 1.19 and Sabogal Hospital with 1.01 cases per 1,000 admissions, respectively. We collected clinical information on 157 isolates. One was deleted due to the lack of data.

### Microbiological results

The frequency of non-*Candida albicans* species was 71.9%. Table 1 shows the distribution of species identified. The most frequently isolated species were *C*. *albicans* (n = 44, 27.8%), *C*. *parapsilosis* (n = 40, 25.3%), *C*. *tropicalis* (n = 39, 24.7%), *C*. *glabrata* (n = 14, 9.5%), and *C*. *guillermondii* (n = 11, 7.0%). Less frequent species included *C*. *lipolytica*, *C*.*lusitaniae* and *C*. *krusei*. All cases were monomicrobial infections, no mixed *Candida species* infections were observed in this series.

**Table 1:** Distribution of *Candida* species by frequency and age group.

*Candida* species	Total	Neonates	Children	Adults	Elderly
*C*. *albicans*	44, 27.8%	5	7	17	15
*C*. *parapsilosis*	40, 25.3%	4	11	15	10
*C*. *tropicalis*	39, 24.7%	1	3	14	21
*C*. *glabrata*	15, 9.5%	0	2	4	9
*C*. *guillermondii*	11, 7.0%	1	3	3	4
*C*. *lipolytica*	6, 3.8%	0	0	1	5
*C*. *lusitaniae*	2, 1.3%	0	0	0	2
*C*. *krusei*	1, 0.6%	0	0	0	1
Total	158, 100%	11	26	54	67

Species distribution according to age group is also shown in Table 1. We did not calculate age-specific incidences, but we observed a large proportion of cases in elderly patients (67/158, 42.4%). The participating institutions have been involved historically in the care of adult and elderly patients, with relatively small pediatric services. This probably accounts for the disparity in age distribution. In this age group, *C*. *tropicalis* was the most frequent species (21/67, 31.3%). *C*. *tropicalis* isolates were predominantly found in elder patients (21/39, 53.8%).

The degree of correlation among the species identified between clinical laboratories at participating hospitals and species identification at the mycology reference laboratory using conventional and molecular methods was 65.8%, while 54/158 isolates were misidentified. In 41 of these 54 isolates, the local laboratories were not able to identify the isolate to species level. This indicates that in 25.9% (41/158) of cases, species identification was not available from the primary clinical laboratory. No other particular pattern of misidentification was observed for the remaining 13 misidentified isolates.

Of the 158 isolates, 86.7% were susceptible to fluconazole as shown in Table 2: four isolates were resistant (1 *C*. *albicans*, 2 *C*. *parapsilosis*, and 1 *C*. *krusei* isolate). Seventeen isolates were classified as susceptible-dose dependent (SDD). This includes 15 *C*. *glabrata* isolates, although all of the MICs for fluconazole were 4 or lower (Table 3). Fluconazole resistant isolates were recovered from patients with high rates of previous antifungal use (2/4, 50.0%), when compared to the susceptible isolates (33/154, 21.4%).

**Table 2:** Frequency of susceptibility to fluconazole (2012 CLSI breakpoints).

Susceptibility to fluconazole	Frequency (n, %)
Susceptible	137, 86.7%
Susceptible dose-dependent (SDD) *	17, 10.7%
Resistant ᵒ	4, 2.6%

*Includes *C*. *glabrata* isolates irrespective of actual MIC

ᵒ Includes 1 isolate of *C*. *albicans*, 2 *C*. *parapsilosis* and 1 *C*. *krusei*

**Table 3:** MIC distribution of antifungal drugs against 5 most common *Candida* species.

TOTALS	≤0.03	0.06	0.12	0.25	0.5	1	2	4	≥8
**Fluconazole**									
*C*. *albicans*			21	15	3	1	2	1	1
*C*. *parapsilosis*			2	15	15	2	1	1	2
*C*. *tropicalis*			10	17	10	2			
*C*. *glabrata*					3	6	4	2	
*C*. *guillermondii*					2	6	2	1	
**Posaconazole**									
*C*. *albicans*	39	2	1	2					
*C*. *parapsilosis*	33	6		1					
*C*. *tropicalis*	29	8	2						
*C*. *glabrata*	6		1	7	1				
*C*. *guillermondii*	3	3	3	2					
**Amphotericin B**									
*C*. *albicans*			3	12	29				
*C*. *parapsilosis*				15	23	2			
*C*. *tropicalis*			3	16	18	2			
*C*. *glabrata*			3	5	7				
*C*. *guillermondii*		4	1	5	1				
**Anidulafungin**									
*C*. *albicans*	43			1					
*C*. *parapsilosis*	4		2	2	12	16	4		
*C*. *tropicalis*	38	1							
*C*. *glabrata*	14				1				
*C*. *guillermondii*	1	1	1		3	5			
**Voriconazole**									
*C*. *albicans*	42		1	1					
*C*. *parapsilosis*	36	2	2						
*C*. *tropicalis*	36		3						
*C*. *glabrata*	10	3	2						
*C*. *guillermondii*	10	1							

The MIC distribution for the most common *Candida* species is shown in Table 3. In addition, all isolates were susceptible to anidulafungin and none had reduced susceptibility to amphotericin B. All *C*. *glabrata* isolates were susceptible to voriconazole and one *C*. *albicans* was SDD for voriconazole. Three *C*. *albicans* strains were detected as non-wild-type for posaconazole (MIC>0.06).

In this study we did not find multi-drug resistance (defined as resistance to two different antifungal drug classes). We identified one isolate of *C*. *albicans* that exhibited cross-resistance (defined as resistance to two antifungals of the same class) against two azoles, and another *C*. *albicans* isolate that was non-wild-type for posaconazole and also showed SDD susceptibility against two azoles (fluconazole and voriconazole).

### Clinical results

The median age of the patients was 55.0 yrs. (0–99), and 100 (64.1%) of them were males. Only 38 (24.2%) of the episodes of candidemia occurred in those subjects < 18 yrs.

The most frequently and most recent (<90 days) associated co-morbid conditions included surgery (n = 61, 38.9%), mechanical ventilation (n = 60, 38.2%) and total parenteral nutrition (n = 57, 36.3%). Frequently identified comorbidities included cancer (n = 34, 21.7%), hemodialysis (n = 14, 8.9%), organ transplantation (n = 8, 5.1%) and febrile neutropenia (n = 8, 5.1%). Isolates were most commonly recovered from the ICUs (n = 55, 35.0%), followed by abdominal surgery wards (n = 53, 33.8%).

Fever was the most commonly encountered clinical finding, observed in 111 cases (70.7%). Clinical presentation syndromes most frequently reported were sepsis (n = 65, 41.4%) and severe sepsis (n = 33, 21.0%). Multivariate analysis showed that mortality was associated with severe sepsis syndrome, which had a relative risk of 3.28 (1.57–6.85, p = 0.001) (Table 4).

**Table 4:** Univariate and multivariate mortality analysis.

	Bivariate			Multivariate		
	Risk Ratio	CI	p-value	Risk Ratio	CI	p-value
**Age**						
Infant (< = 1 yr)	1	Ref.		1		
Children (1 to 18 yrs)	0.29	(0.64–1.33)	p = 0.112	0.45	(0.09–2.24)	p = 0.334
Adult (19 to 60 yrs)	0.71	(0.31–1.62)	p = 0.418	0.50	(0.19–1.34)	p = 0.171
Elderly (> 60 yrs)	1.08	(0.52–2.23)	p = 0.834	0.76	(0.31–1.87)	p = 0.552
**Hospital**						
Almenara	1			1		
Dos de Mayo	0.74	(0.31–1.77)	p = 0.511	0.67	(0.26–1.72)	p = 0.413
Sabogal	1.67	(0.84–3.29)	p = 0.139	0.95	(0.38–2.37)	p = 0.918
**Sex**						
Female	1	Ref.		1		
Male	1.16	(0.65–2.06)	p = 0.597	1.04	(0.54–1.97)	p = 0.913
***Candida* species**						
*C*. *albicans*	1	Ref.		1		
*C*. *parapsilosis*	0.70	(0.34–1.41)	p = 0.322	0.90	(0.41–1.99)	p = 0.801
*C*. *tropicalis*	0.78	(0.37–1.64)	p = 0.519	1.04	(0.45–2.43)	p = 0.920
Other	0.62	(0.28–1.40)	p = 0.247	0.73	(0.29–1.83)	p = 0.504
**Pre-existing conditions**						
None	1			1		
One	0.75	(0.30–1.88)	p = 0.543	0.57	(0.22–1.54)	p = 0.274
Two or more	1.01	(0.42–2.47)	p = 0.967	0.61	(0.23–1.61)	p = 0.320
**Karnofsky score**						
< = 40	1			1		
> = 50	0.17	(0.04–0.70)	p = 0.015	0.22	(0.05–1.00)	p = 0.051
**Antifungal treatment**						
Not treated	1			1		
Treated	0.49	(0.28–0.85)	p = 0.012	0.40	(0.20–0.79)	p = 0.009*
**Severe sepsis**						
No	1			1		
Yes	3.46	(1.96–6.1)	p = 0.000	3.28	(1.57–6.85)	p = 0.001*

* Statistically significant.

Forty-four (28.6%) patients received antifungal treatment within 72 hrs. of clinical suspicion of candidemia, as indicated by blood culture request. In the remaining cases, treatment was started ≥ 72 hrs. after blood culture (n = 55, 35.0%), or was not started at all (n = 58, 36.9%). Early mortality (<7 days) explained no initiation of treatment in 23 cases. For the remaining 35 cases, no initiation of antifungal therapy was more frequent when the patient was not in an ICU (25/35, 71.4%), and more likely at Dos de Mayo Hospital (13/30, 43.3%) and Sabogal Hospital (7/29, 24.1%), compared to Almenara Hospital (15/98, 15.3%).

Fluconazole was the most frequently prescribed antifungal (61.5%), followed by amphotericin B (23.3%). No differences in the patterns of prescription were observed across different groups of patients (by age, ward, and hospital). Only (16.2%) of subjects received caspofungin.

**Fig 1:**
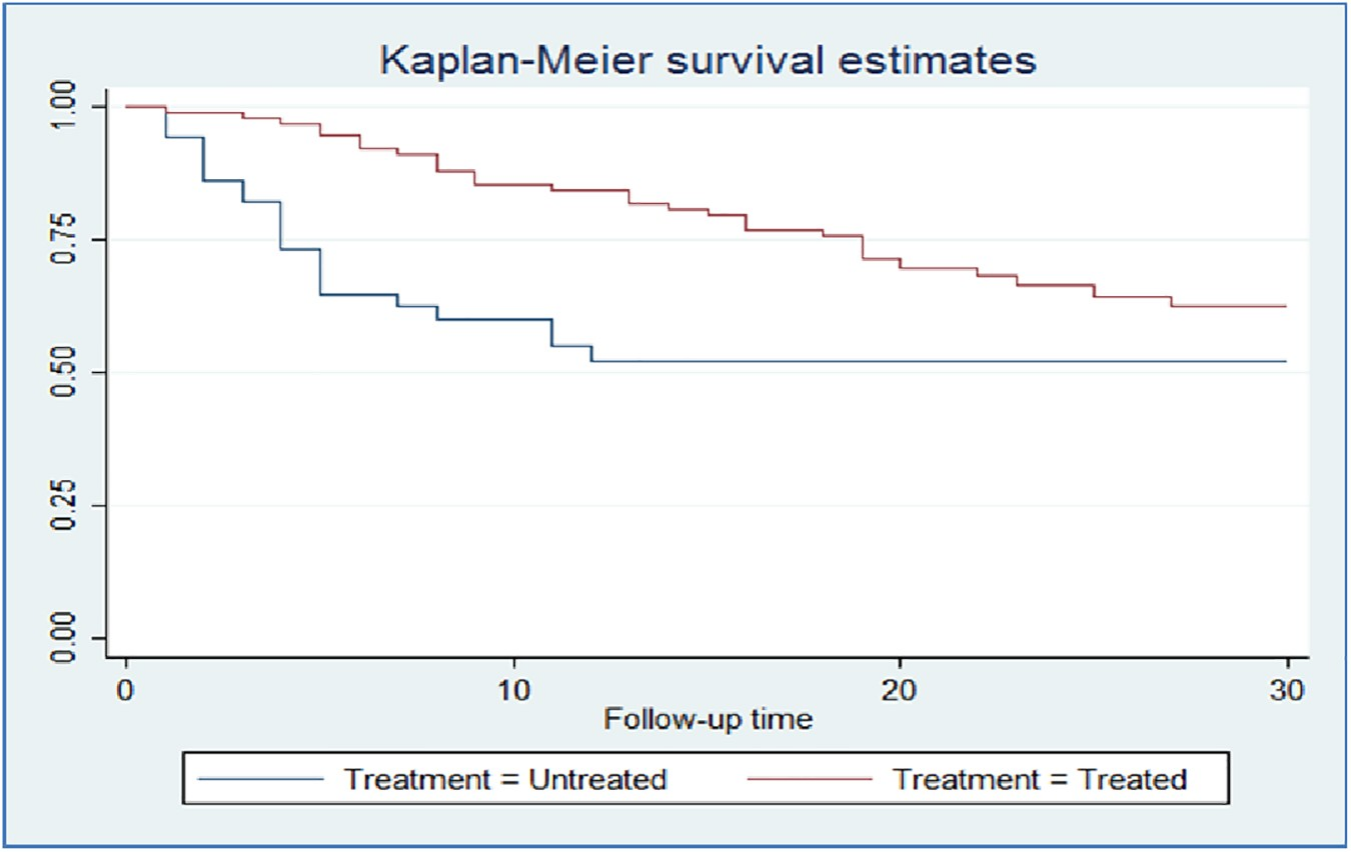
30-day survival curve by treatment of candidemia (RR vs. days).

Survival at day 30 after culture positivity was 60.4%, and was higher for patients that received antifungal therapy (67.4%) when compared to patients that did not receive an antifungal (50.9%). The overall mortality rate was 24.8 deaths per 1000 patient-days. Antifungal therapy had a beneficial effect on survival with a relative risk of death of 0.40 versus patients that did not receive antifungal therapy (0.20–0.79, p = 0.009) (Fig 1).

Using univariate and multivariate analysis, mortality was not associated to age group, gender, participating Hospital, *Candida* species or, in the case of patient with multiple comorbidities, with the number of pre-existing conditions (1, 2 or >3) (Table 4).

Mortality was not significantly different in the extremes of age, although it was lowest in the elderly patients (52.4% 30-day survival). The 30-day survival for *C*. *albicans* candidemia was 52.6%, slightly lower than the survival rate of 59.2% in candidemia due to non-*albicans Candida* species. The difference did not reach statistical significance (Table 4).

## Discussion

Our results show a high rate of candidemia in these three Peruvian hospitals, which appears to be higher than the highest country incidence described by Nucci et al [2] in a recent epidemiological survey of Latin American countries. In fact, the present study has an aggregate rate of 2.04 vs. 1.95 episodes/1000 admissions for Argentina, and 1.18 as a global result for the rest of Latin America. In Nucci’s study data was collected from 21 hospitals in 7 countries. It did not include Peruvian hospitals. Furthermore, data from industrialized countries has demonstrated a lower incidence and stable trends for candidemia rates, which are closer to 1.0 case/1000 admissions [6–10]. Recent Italian studies by DeRosa et al. [20] and Tedeschi et al. [21] reveal an incidence close to the Latin American reports (1.8–2.2/1000 admissions).

In contrast, a recent report from China also showed lower rates of candidemia in Shanghai. [22] In a separate epidemiologic study, a survey from a trauma center in India reported an overall incidence of candidemia of 7.76 and of 14.95 for ICU admissions, the highest incidence rates reported to date. [23]

In recent years, candidemia has become a more frequent cause of nosocomial infections in Peruvian hospitals. This is especially true when populations of immunocompromised individuals or complex cases with associated multiples comorbidities are taken care of with limited access to critical care facilities. In this series, most cases were identified outside of the ICUs (65.0%), similar to the most recent trends observed in other Latin American countries. [24]

Although *C*. *albicans* was the most frequently recovered *Candida* species, we describe a very high proportion of non- *Candida albicans* species (71.9%), with *C*. *parapsilosis* and *C*. *tropicalis*, being almost as common as *C*. *albicans*. *C*. *glabrata* was the fourth most frequently isolated Candida species (8.9%), followed by *C*. *guillermondii*, for 93.5% of all of the isolates recovered. In this series we found only one *C*. *krusei* isolate.

In general, about 92–96% of cases of candidemia are caused by five species (*Candida albicans*, *C*. *glabrata*, *C*. *tropicalis*, *C*. *parapsilosis*, *and C*. *krusei*). [24, 25].

The results from this study are similar to the results reported in the different candidemia studies by Godoy et al. [26] and dos Santos et al. [27] in Brazil, Riera et al. [28] in Argentina, by Cortés et al. [1] in Colombia, Bustamante et al. [4] in a different group of Lima hospitals, and the large Latin American series reported by Nucci et al.[2] It appears that *C*. *krusei* is an species not frequently isolated in Latin American countries, as well as in the Asia-Pacific regions.[29]

Decreased susceptibility/resistance to fluconazole in the entire sample was 13.3% (n = 21), primarily due to *C*. *glabrata* isolates (n = 15), although in our series, it did not show reduced susceptibility.

The absence of isolates resistant to anidulafungin and the low percentage of reduced susceptibility to voriconazole and posaconazole may be due to the limited use of these antifungal drugs during the study period. Similar results have been described in the candidemia study from other Latin American countries.[2] In this large study all isolates were susceptible to voriconazole, and no isolates were resistant to anidulafungin, although 5 out of 653 of isolates had intermediate susceptibility. Susceptibility monitoring of these drugs and evaluation of their clinical significance in the near future will be very important as use of these drugs will likely increase in the region.

The median age of the candidemic patient was higher in this series than in other series evaluating candidemia in Latin America. This could be influenced by the older age group of the majority of our patients. The median age found in this series was 55.0 yrs., much higher than the age reported by Nucci (26.0 yrs.), [2] Cortés (41.2 yrs.)[1] and Wille (32.4 yrs.)[3]. However, the age was similar to the median age (56.0 yrs.) reported more recently from a multi-regional surveillance study of candidemia in Brazil. [30]

Furthermore, the cases seen in the elderly population in this series also demonstrated a high mortality and an association with predisposing co-morbid conditions such as cancer, and is similar to what was described in a series specifically focused on evaluating candidemia in older individuals. [31, 32]

In this study, the overall survival rate on day 30 was 60.4% (treated subjects, 67.4%; not-treated subjects 50.9%), approximately similar to recent reports [21, 33, 34], with an overall mortality rate of 24.8 deaths per 1000 patient-days. In addition, the younger patients in this series had a lower survival rate than that reported by Santolaya et al. [35] for a Latin American series evaluating the pediatric patients (60 vs. 72% survival at 30 days.).

Risk factors for invasive candidiasis and candidemia included *Candida* colonization, severe illness, exposure to broad-spectrum antibiotics, recent major surgery, necrotizing pancreatitis, dialysis, parenteral nutrition, corticosteroids, and the use of CVCs. [36] We did not collect information on colonization. However, other known risk factors described previously were very common in our study population. Although this study did not attempt to identify risk factors, it did reveal that known risk factors for developing candidemia were commonly seen in our patients.

In this series, most patients were treated with either fluconazole or amphotericin B. In this country, it remains to be seen whether an increased utilization of echinocandins improves outcomes or is associated with increased resistance.

In many countries, Peru amongst them, candidemia is not recognized as an important and serious cause of sepsis. A non-specific clinical syndrome and a low clinical suspicion in patients at high-risk appear to be important factors that contribute to the delay in the diagnosis and the delayed initiation of appropriate antifungal therapy.

Delay in the initiation of antifungal therapy was probably associated with the elevated mortality rate, in spite of relatively low antifungal resistance rates. Moreover, our data showed a beneficial effect of initiation of antifungal therapy, since it was associated with a decrease in mortality. Earlier initiation of antifungal therapy has been recognized as a beneficial predictor of survival. [21, 37] No indication of antifungal therapy was associated to early mortality, and also to location of patients (out of ICU, hospitals with lower incidence of candidemia). Increased awareness and an active surveillance are necessary for improving outcomes of patients with candidemia.

Limitations of our study include a relatively small sample to evaluate incidence of candidemia for specific sub-groups of patients (age or diagnosis), and a period of observation insufficient to describe temporal trends. A more prolonged period of observation and a different study design would be required for this objective. [38]

## Conclusions

The incidence of candidemia in these Peruvian hospitals was high, at 2.04 cases per 1000 admissions. An unusually high percentage of our *Candida* species were non-*Candida albicans* species, with *C*. *parapsilosis* and *C*. *tropicalis* being almost as frequent as *C*. *albicans* Fluconazole resistance remains very low at 12.7%, including the *C*. *glabrata* isolates. In spite of low antifungal resistance rates, this study shows a high mortality rate. Very possibly, the delay in the initiation of antifungal treatment probably contributes to the elevated mortality rate described in this study.

## Supporting information

S1 File Candidemia in Lima, Peru. Microbiological information.
(XLSX)

S2 File Candidemia in Lima, Peru. Clinical information.
(XLSX)
